# Nurses’ Knowledge of Rare Diseases: A Systematic Review

**DOI:** 10.3390/nursrep15090321

**Published:** 2025-09-04

**Authors:** Inmaculada Muñóz Sánchez, Jose Manuel Martínez-Linares, Raquel Rodríguez-Blanque, Jonathan Cortés-Martín, Andrés Reinoso-Cobo, Beatriz Lechuga Carrasco, Juan Carlos Sánchez-García

**Affiliations:** 1 Gran Capitán Health Center, Granada Health District, 18013 Granada, Spain; inmams1@correo.ugr; 2 Department of Nursing, Faculty of Health Sciences, University of Granada, 18071 Granada, Spain; jmmartinezl@ugr.es (J.M.M.-L.); rarobladoc@ugr.es (R.R.-B.); jsangar@ugr.es (J.C.S.-G.); 3 Department of Nursing and Podiatry, Faculty of Health Sciences, University of Malaga—Teatinos, Arquitecto Francisco Peñalosa 3, 29071 Malaga, Spain; andreicob@uma.es; 4 Virgen de las Nieves University Hospital, 18014 Granada, Spain; beatriz.lechuga.sspa@juntadeandalucia.es

**Keywords:** rare diseases, nursing, level of knowledge, health literacy

## Abstract

**Background**: Rare diseases affect fewer than 1 in 2000 people, but collectively, they impact millions. Their diagnosis and management present challenges due to low prevalence, clinical heterogeneity, and a lack of standardized protocols. Nurses play a key role in assisting and caring for these patients by providing direct care, emotional support, and health education. **Objective**: The objective of this systematic review is to update the existing knowledge on nurses’ level of understanding regarding rare diseases, as a decline in their training can compromise the quality of care and access to early detection. **Methodology**: A bibliographic search was conducted in Scopus, PubMed, CINAHL, SciELO, and Cochrane Library, selecting studies published between 2014 and 2024 on rare disease knowledge. The PRISMA model was followed, and the review was registered with PROSPERO under code CRD42024580656. **Result**: Ultimately, 24 studies were included. The main results showed a significant gap in nursing education concerning rare diseases. **Conclusions**: Continuous education, telemedicine, and the integration of health technologies were highlighted as improving competencies in rare diseases. Therefore, it is a priority to increase nursing training in rare diseases at all levels.

## 1. Introduction

### 1.1. Background

Rare diseases, also known as infrequent or orphan diseases, represent a significant challenge in public health due to their low prevalence and a general lack of widespread knowledge. These pathologies, affecting a small proportion of the population, exhibit significant heterogeneity in their clinical manifestations and treatment needs. Globally, it is estimated that there are between 6000 and 8000 rare diseases, collectively affecting approximately 300 million people worldwide. Although each of these diseases generally has a low prevalence (affecting fewer than 1 in 2000 people), their combined impact poses a major medical and social challenge due to diagnostic complexity, limited knowledge among healthcare professionals, and a scarcity of effective treatments [1,2].

In Europe, the prevalence of these diseases is similar, with an estimated 30 million people affected, according to the European Organization for Rare Diseases (EURORDIS). This organization highlights that the management of these pathologies in Europe requires a collaborative, interdisciplinary approach involving researchers and public health policies that promote the establishment of care networks and specialized centers. Within the European Union, a rare disease is defined as one affecting fewer than 5 people per 10,000, making cross-country cooperation essential for research and treatment [2].

In Spain, estimated figures indicate that approximately three million people suffer from a rare disease. This context underscores an urgent need for adequate training and resources, particularly within the nursing field, where direct patient care is essential. In this regard, national rare disease associations and health policies are moving toward greater visibility and support for these patients, although significant challenges persist [3].

The role of nurses is fundamental in the management of patients with rare diseases, as nurses often provide direct care, emotional support, and health education to both patients and their families. However, the limited knowledge of these pathologies among nurses constitutes a significant barrier to providing optimal care. Specific training in rare diseases is scarce, and most educational programs do not include specialized content in this field. This gap in formal education contributes to diagnostic uncertainty, a lack of skills for adequate clinical management, and ultimately, a lower quality of care for patients suffering from these diseases [4]. In this context, the key question guiding this review arises: what is the level of knowledge of rare diseases possessed by nurses?

This problem is exacerbated by the complex and frequently multisystemic characteristics of rare diseases, which demand a multidisciplinary and specialized approach. Nurses, being in close contact with patients, are in a privileged position to detect symptoms, identify specific needs, and coordinate care with other health professionals. However, this competence can only be effectively exercised if they possess the necessary knowledge to recognize the particularities of rare diseases and understand their clinical and social implications [5].

Nurses’ knowledge of rare diseases is a topic that has gained relevance in recent years due to the increasing need to improve care and the early diagnosis of these pathologies [6]. As rare diseases affect a small proportion of the population, they tend to be poorly understood among healthcare professionals, which impacts the quality of care patients receive. The scientific literature has begun to address this issue, highlighting the importance of specialized nursing education [7].

The necessity for nurses to be trained in the approach to rare diseases is emphasized, given its direct influence on the quality of care and the health outcomes of affected patients [8,9,10].

### 1.2. Objective

The objective of this systematic review is to update the existing knowledge regarding nurses’ understanding of rare diseases.

## 2. Methodology

### 2.1. Review Protocol

The methodology used for this report was a systematic review of the scientific literature on nurses’ knowledge of rare diseases. We followed the Preferred Reporting Items for Systematic Reviews and Meta-Analyses (PRISMA) protocol, which consists of a 27-item checklist covering the most representative sections of an original article, as well as their development process. This review was registered in the PROSPERO international prospective register of systematic reviews 2024 with the code CRD42024580656, available at https://www.crd.york.ac.uk/prospero/display_record.php?ID=CRD42024580656 (accessed on 1 July 2025).

### 2.2. Eligibility Criteria

We selected articles published between 2014 and 2024 that presented information on nurses’ knowledge of rare diseases. No restrictions were placed on the language of publication or the research design of those articles.

### 2.3. Information Sources

The bibliographic search was conducted in the Scopus, PubMed, CINAHL, SciELO, and Cochrane Library databases. A manual search was also performed using reference lists from the included studies to identify additional relevant articles.

The structured language for the search was derived from Medical Subject Headings (MeSH) terms and Health Descriptors (DeCS). The MeSH terms used were “Rare Diseases”, “Nursing”, and “Level of Knowledge”. The Boolean operators OR and AND were utilized.

### 2.4. Search Strategy

The following table (Table 1) presents the search strategy used for this systematic review.

### 2.5. Data Extraction Process

Following the implementation of the search strategy, from June to July 2025, the identified articles were transferred to the Mendeley web application using Mendeley’s web importer tool. Subsequently, they were organized into folders based on their source database, and all duplicates were removed.

Articles on nurses’ knowledge of rare diseases, published between 2014 and 2024, were included. Two reviewers (IMS and JCM) extracted data from the title, abstract, and keywords of each identified study. These reviewers independently examined each study and applied the inclusion and exclusion criteria.

For potentially eligible studies, the same procedure was applied to full-text articles. Any queries about a particular article were resolved through discussion with the project supervisor (JCM). Data on quality, patient characteristics, interventions, and relevant outcomes were extracted by IMS under the supervision of the project supervisor (JCM). Discrepancies between reviewers were resolved by a third reviewer.

### 2.6. Data Collection Process and Collected Data

Two reviewers extracted the most relevant data from each article, including authorship, publication year, article title, study objectives, and obtained results. The strengths and weaknesses of each study were also assessed. The Results section provides a more detailed explanation of the article selection process.

### 2.7. Risk of Bias in Studies

To evaluate the methodological quality of the selected articles, a structured analysis was conducted based on the design and methodology of each study. It is true that articles sometimes have structures that blend several research designs. To classify these articles and perform the methodological evaluation, we focused on the dominant design and used the relevant scale. Of the 24 articles selected for this systematic review, 3 were systematic reviews [11,12,13], 2 were clinical practice guidelines [14,15], 7 were observational and descriptive studies [16,17,18,19,20,21,22], 4 were qualitative studies [23,24,25,26], 1 was a case study [27], 2 were mixed-methods studies [28,29], and 5 were non-randomized intervention or implementation studies [30,31,32,33,34]. Each group was evaluated using a specific scale to determine its methodological rigor and associated risk of bias.

The methodological quality of the systematic reviews (Table 2) was evaluated using the AMSTAR-2 Scale (Assessing the Methodological Quality of Systematic Reviews). Through 16 domains assessing methodological rigor, transparency, and replicability, studies were classified as high, moderate, low, or critically low confidence. Of the selected studies, most exhibited moderate to high confidence, with only 2 reviews rated at a low level.

The AGREE II scale was used for the clinical practice guidelines (Table 3), which is a tool for evaluating the methodological quality of clinical practice guidelines. It serves to assess the rigor of their development, the clarity of the recommendations, and the management of conflicts of interest, allowing reviewers to determine if a guideline is reliable and applicable. The scoring is based on 23 items grouped into 6 domains, with a final rating that helps reviewers decide whether the guideline is recommended for use.

To evaluate the methodological quality of descriptive studies, the STROBE scale (STrengthening the Reporting of Observational studies in Epidemiology) was used (Table 4). It is not a scoring scale, but a 22-item checklist. Its main objective is to improve the quality of reporting for observational studies, such as cohort, case–control, and cross-sectional studies.

For the articles with a qualitative design, the CASP (Critical Appraisal Skills Programme) scale was used. It is a checklist designed to assess the quality of qualitative research. Unlike tools that score numerically, CASP uses a series of questions to guide the evaluator in assessing the rigor, relevance, and credibility of a qualitative study. The methodological quality of the qualitative studies [23,24,25,26] was evaluated with this scale. In general, the studies showed good rigor in describing the research objectives and in selecting participants, which strengthens the credibility of their findings. However, weaknesses were identified in the discussion of the relationship between the researcher and participants, and in the validation of findings with participants. Although these methodological limitations were observed in some articles, they did not affect the internal validity of the body of evidence. The results are considered sufficiently rigorous to be included in this review, as they provide a deep understanding of the experiences and perspectives of caregivers and nurses.

The CARE (CAse REport) scale is a 13-item checklist designed to improve the quality of case reports in the medical literature. The main purpose of the CARE checklist is to ensure that the authors of case reports include all the necessary information so that the reader can assess the validity of the case and its findings. A well-documented case report can be invaluable to medical knowledge, especially in the context of rare diseases, new disease presentations, or unusual side effects. This scale was used to evaluate the methodological quality of the clinical case report by Medina-Ortega et al. [27], which included 11 out of the 13 items proposed in this scale.

The articles by Morris et al. [28] and Guccione et al. [29] are mixed-methods articles; the MMAT scale was used for them. The MMAT (Mixed Methods Appraisal Tool) scale is a tool used to evaluate the methodological quality of studies that combine different research designs, including qualitative, quantitative, and mixed-methods studies. Its purpose is to determine the reliability of a study’s conclusions that integrate multiple methods. It does not assign a numerical score; instead, it assesses the rigor of each component with a “Yes”, “No”, or “Can’t tell” response, allowing for a descriptive analysis of the study’s strengths and weaknesses. The study by Morris et al. [28] demonstrates high methodological quality in its mixed-methods design. The qualitative and quantitative components are well-defined, and the integration between them is logical and strengthens the findings. While there might be limitations in the quantitative component (such as the response rate), the rigor and coherence of the overall design make its results reliable for the purposes of this review. The protocol by Guccione et al. [29] demonstrates high methodological quality in its design, despite being an early-phase study. Although its quantitative design is not randomized (which would be a weakness in a later-phase trial), it is appropriate for the study’s objective (feasibility and safety). The integration of the qualitative and quantitative components is solid and well-justified, suggesting that the study, once conducted, could yield reliable findings.

Finally, for non-randomized intervention or implementation studies, the TREND scale was used (Table 5). The TREND (Transparent Reporting of Evaluations with Nonrandomized Designs) scale is a 22-item checklist designed to improve the quality and transparency of reporting for intervention or implementation studies that do not use randomization. The main goal of the TREND checklist is to ensure that authors of non-randomized studies present all the necessary information so that other researchers and healthcare professionals can evaluate the validity of their results.

## 3. Results

**Figure 1:**
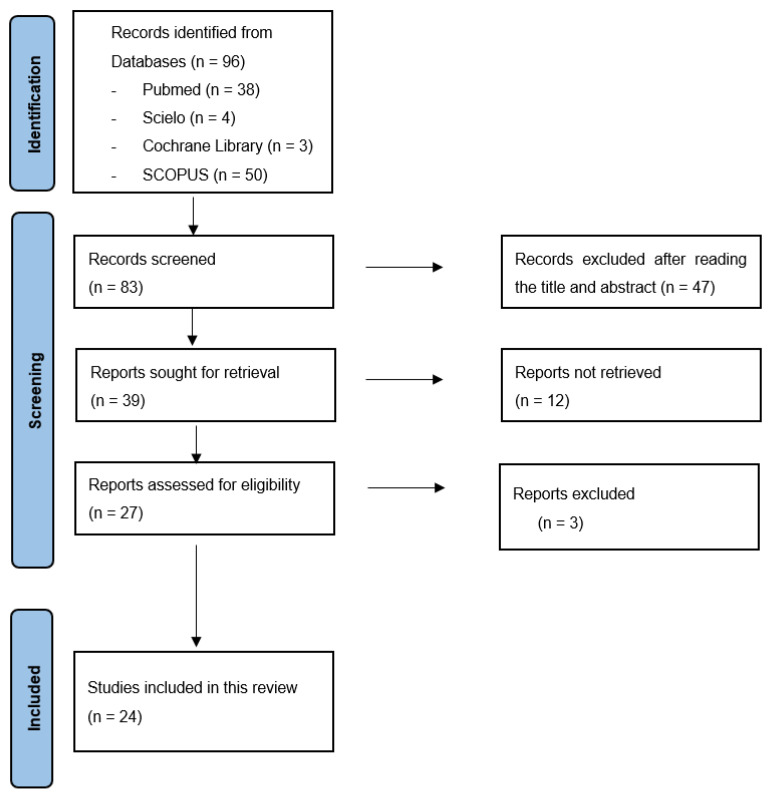
Flow diagram.

The flowchart of this systematic review is presented below (Figure 1).

The following table (Table 6) presents a summary of the main results.

Of the 24 articles selected for this systematic review, 45.83% was from Europe [12,13,14,15,16,18,27,28,30,31,34]; 8.3% was from the USA [32,33]; the same percentage, 8.3%, was from Australia [11,29]; 12.5% was from the Asian continent [17,21,22]; and finally, 25% was from South America [19,20,23,24,25,26]. It is certainly difficult to study the origin of an article since many cases are reports with shared authorship. For this reason, this classification was based on the affiliation of the first author of each article.

The analyzed studies reveal a significant gap in nursing education concerning rare diseases. Walkowiak et al. assessed nursing knowledge in Poland, with 120 nurses and 150 nursing students participating. Their findings showed that 75% of nurses and 85% of students did not feel prepared to care for patients with rare diseases [16]. Another study by Gómez-Díaz et al. in Mexico, which designed a training program, included 200 students and professionals and found that the percentage of correct answers in a rare disease knowledge evaluation increased from 38% before the intervention to 93% after training [20]. Awareness and knowledge were also explored in the study by Sinan et al., who examined awareness and knowledge among 350 healthcare professionals in a tertiary hospital in Bahrain. They found that 87.4% of respondents recognized the term “rare diseases”, although their training in this area was limited [17]. Similarly, Jahanshahi et al., studying the perception of rare disease knowledge among 180 future healthcare professionals in Iran, found that 72% of participants did not feel prepared to care for these patients, despite 70% having received some content on these pathologies during their university education [22]. The importance of training quality has also been examined, revealing differences in the availability of such content across regions. A study by Claahsen et al. evaluated multiple registries that examined the integration of training modules on rare diseases into nursing curricula in several European countries [15].

Another aspect emphasized by some of the analyzed studies pertains to interventions in care models. Pleutim et al. explored practices based on person-centered care models for Huntington’s disease from the perspective of family caregivers, focusing on adapting care environments and specialized training. Online rare disease training modules were utilized, and participation in webinars and self-study modules reportedly increased the understanding of these pathologies by 78% [26]. In this context, Ward et al. demonstrated that the integration of nurses into multidisciplinary teams and their role in care coordination were essential, following an evaluation of their participation in interprofessional teams in Ireland. They documented that collaboration with social workers and psychologists fosters better organization of care for patients with rare diseases [34]. Similarly, Morris et al. examined the access of 760 patients with rare diseases to specialized services in the UK and found that only 12% of patients had a formal care coordinator, and 32% attended a specialized center [28].

Regarding nurses’ roles in patient and family education, a study by Kis et al. analyzed the operation of a nurse-led telephone counseling line, through which 300 calls were made to patients with rare rheumatological diseases. It showed that 61% of inquiries were related to appointment scheduling, 33% to specialist referrals, and 7% to urgent care [30]. In the same vein, Martínez Reyes et al. examined the needs of 150 caregivers of individuals with rare diseases and concluded that nurses play a key role in providing information on symptomatic management, treatment administration, and access to psychosocial support [24]. The importance of professional support and patient well-being is highlighted in studies like that by De Souza et al., where 200 patients with autoimmune diseases were selected to assess the impacts of emotional and social support on their quality of life [23]. Additionally, the absence of specialized knowledge limits the guidance and support that professionals can offer caregivers, as reflected in the study by Pelentsov et al. This study evaluated the support needs of parents caring for children with rare diseases. Key findings included social needs (72% of parents expressed social isolation due to lack of adequate support networks), information needs (65% reported difficulty accessing reliable, specific information about their children’s disease), and emotional needs (62% reported high levels of stress and anxiety related to diagnostic uncertainty and nurses’ lack of preparedness to address these pathologies) [11].

Finally, concerning the applications of new technologies, Pinto et al. assessed the impact of integrating genomics into clinical practice for 1152 patients through five-week programs that utilized simulation and collaboration with geneticists. They reported a 17.5% increase in diagnostic resolution rates, reaching up to 66.7% for certain phenotypes [33]. Similarly, the work by Ferreira et al. studied the use of telemedicine in primary healthcare for rare diseases. Including 78,000 patients, they found that 20- to 30-min virtual consultations facilitated early diagnosis and improved the continuity of patient follow-up [31]. Additionally, a study by Herbert et al., which implemented a hybrid training program on genetics and differential diagnosis involving 85 nurses, found that the most relevant result was that 85% of participants improved their ability to identify clinical signs of rare diseases, and 67% expressed greater confidence in their clinical practice [25].

## 4. Discussion

The literature analysis reveals that the care of patients with rare diseases faces significant structural challenges that go beyond mere access to diagnostics and treatments. The main conclusion of this systematic review is that the absence of standardized training and the scarcity of specific educational strategies for nursing staff not only affect the clinical response but also perpetuate inequalities in access to specialized care. This training gap, identified by studies such as those by Walkowiak et al. and Sinan et al. [16,17], shows a marked heterogeneity that results in significant deficiencies in clinical management and patient education.

The lack of knowledge and specialization has a direct impact on patients’ perception of care quality. Bogart et al.’s findings [8] suggest that the perception of deficient care can negatively influence therapeutic adherence and psychosocial well-being. This perspective is reinforced by Pelentsov et al. and Sousa et al. [11,23], who document that the lack of professionals with specific training creates a deep sense of isolation for patients and their families, who often face additional barriers in finding support and guidance. Gómez-Díaz et al. [20] further add that this lack of training contributes to care fragmentation, creating variability in care depending on the institution, which increases the vulnerability of these patients.

Despite these barriers, the literature also offers promising solutions. Vicente et al. [7] and Kis et al. [30] demonstrate that continuous training in rare diseases and the implementation of innovative care interventions, such as nurse-led telephone triage lines, can improve early detection capabilities and optimize the management of complex cases.

At the level of health policies, this review highlights the lack of a global consensus on nursing training in rare diseases. Czech et al. [12] point out that the absence of specific training programs, the lack of reliable epidemiological registries, and disparities in access to orphan treatments remain critical barriers to equitable care. Tsitsani et al. and Jahanshahi et al. [13,22] complement this view by arguing that if training in rare diseases is not integrated into formal nursing education, the healthcare system’s capacity to respond to these pathologies will remain limited.

However, the growing integration of digital health and genomic medicine in the management of rare diseases presents an opportunity to overcome these barriers. Studies by Pinto et al. and Ferreira et al. [31,33] suggest that the use of advanced clinical databases and telemedicine can contribute to better identification of clinical patterns, facilitating a more comprehensive and personalized approach. To leverage these advances, the discussion focuses on the need for nursing training programs to include specific content on these technologies.

### 4.1. Limitations

Despite the rigorous selection and analysis process, this systematic review has certain limitations that should be considered. The main one is the scarcity of specific publications on nursing education for rare diseases, which restricted the sample size and, therefore, the generalizability of the findings. Additionally, a notable variability in the research designs of the included articles was identified, ranging from observational and qualitative studies to clinical guidelines and case reports. This methodological heterogeneity makes direct comparison between studies difficult and could influence the robustness of the conclusions, as the evidence does not come from a single, standardized research design.

### 4.2. Future Research Areas Derived from This Study

A priority research area for the future could be the validation of tools to assess nurses’ knowledge of rare diseases. The design of a specific questionnaire addressing this aspect would not only allow for quantifying existing training gaps but also for evaluating the effectiveness of specific educational interventions.

Likewise, the development of longitudinal studies is recommended to analyze the impact of rare disease training on the quality of care and patient experience, allowing for the establishment of evidence-based strategies for the continuous improvement of care in this field.

### 4.3. Implications for Clinical and Academic Practice

The findings of this systematic review reveal the urgent need to address knowledge gaps in nursing regarding rare diseases.

-Continuing Education and Protocols: It is crucial to implement continuing education programs and standardized protocols. This improves the ability of professionals in early detection, clinical management, and care coordination.

-Reducing Inequities: Strengthening the role of nursing through specialized training helps reduce care fragmentation and inequities in care, which translates into improved therapeutic adherence and patient well-being.

-Validation of Instruments: Validation of standardized evaluation tools is necessary to objectively measure nursing staff’s knowledge of rare diseases. This will allow for quantifying knowledge gaps and evaluating the effectiveness of future educational interventions.

-Longitudinal Studies: Longitudinal studies are recommended to analyze how training in rare diseases translates into tangible improvements in care quality and patient experience over the long term.

## 5. Conclusions

This systematic review highlights a critical need to improve training and available resources for healthcare professionals, particularly nurses, in the management of rare diseases.

A lack of specific knowledge and protocols is identified, along with the limited availability of reliable information due to the scarcity of registries in this regard. These factors affect the quality of care and generate uncertainties at multiple levels, from diagnosis to treatment and patient support.

It is essential that undergraduate nursing education programs include content on rare diseases and promote evidence-based research, as well as the implementation of specific training courses.

It is considered necessary to promote collaborative initiatives among healthcare institutions, academic bodies, and patient associations. Furthermore, the integration of new technologies could offer promising solutions to reduce these uncertainties, provided they are implemented within an appropriate regulatory framework. Improving the training and support for healthcare professionals in this area will not only benefit patients but also contribute to more equitable and efficient care within the healthcare system.

## Figures and Tables

**Table 1:** Search string.

Database	Search String
**SCOPUS**	((TITLE-ABS-KEY (“rare diseases” OR TITLE-ABS-KEY (“orphan diseases”) AND TITLE-ABS-KEY (“knowledge”) OR TITLE-ABS-KEY(“awareness”) AND TITLE-ABS-KEY(“nurses”) OR TITLE-ABS-KEY (“nursing”)
**PUBMED**	Search: ((“rare diseases” (MESH) OR “orphan diseases” (MESH) OR “rare diseases” (Title/Abstract) AND (“knowledge”) (MESH)) OR (“awareness” (MESH)OR “perception) ((MESH)) OR “knowledge” (Title/Abstract) OR “awareness” (Title/Abstract) OR “perception” (Title/Abstract)) AND (“nurses”) (MESH) OR “nursing” (MESH) OR “nurses” (Title/Abstract) OR “nursing” (Title/Abstract))
**SCIELO**	TS = (“rare diseases” OR “orphan diseases”) AND TS = (“knowledge” OR ”awareness” OR ”perception”) AND TS =(“nurses” OR ”nursing” OR “health professionals”)
**CINAHL**	TI (“rare diseases” OR “orphan disease”) AND AB (“knowledge” OR “awareness” OR “perception”) AND MH (“nurses” OR “nursing”)
**COCHRANE LIBRARY**	TI (“rare diseases” OR “orphan diseases”) AND TI (“knowledge” OR “awareness”) AND TI (“nurses” OR “nursing”)

**Table 2:** Methodological evaluation of systematic reviews (AMSTAR-2 Scale).

Article	Score
Pelentsov et al. [11]	Moderate
Czech et al. [12]	Moderate
Tsitsani et al. [13]	High

**Table 3:** Methodological evaluation of clinical practice guidelines (AGREE II).

Article	Score
Mantovani et al. [14]	6.8 Strongly recommended
Claahsen et al. [15]	5.3 Recommended

**Table 4:** Methodological evaluation of descriptive studies (STROBE).

Article	Score
Walkowiak et al. [16]	17/22
Sinan et al. [17]	19/22
Lozano et al. [18]	21/22
Figueiredo et al. [19]	20/22
Gómez- Díaz et al. [20]	15/22
Günner et al. [21]	16/22
Jahanshahi et al. [22]	20/22

**Table 5:** Methodological evaluation of non-randomized intervention or implementation studies (TREND).

Article	Score
Kis et al. [30]	15/22
Ferreira et al. [31]	17/22
Kamm et al. [32]	20/22
Pinto et al. [33]	14/22
Ward et al. [34]	16/22

**Table 6:** Results table.

Author	Date	Article Title	Objective	Result	Conclusion
Walkowiak et al. [16]	2020	Needs assessment study of rare disease education for nurses and nursing students in Poland	To analyze the knowledge and opinions of rare diseases among Polish nurses and nursing students	Neither group felt well-prepared to deal with patients with rare diseases, with 75% of nurses and up to 85% of nursing students expressing concern.	Both nurses and nursing students show insufficient knowledge of rare diseases.
Sinan et al. [17]	2023	Knowledge and Awareness of Rare Diseases Among Healthcare Professionals in the Kingdom of Bahrain	To assess the general awareness and knowledge of healthcare professionals regarding rare diseases in a tertiary hospital in the Kingdom of Bahrain	Most participants (87.4%) were aware of and had heard the term “rare diseases” before this survey.	This study highlights the need to improve rare disease knowledge among healthcare professionals, aligning with the global knowledge landscape.
Kis et al. [30]	2019	A pilot nurse-led telephone triage line of patients with rheumatologic rare diseases.	To evaluate the impact of a nurse-led telephone line in managing patients with rare rheumatologic diseases	Improvements in care coordination occurred (61% of calls related to scheduling, 33% were referred to specialists, and 7% were handled by emergency services).	Importance of continuous training in triage for specialized nurses and communication skills to address uncommon diseases
Lozano et al. [18]	**2021**	Inclusive Educational Best Practices for Students with Rare Diseases.	To identify successful educational strategies for the inclusion of students with rare diseases	The teacher–student relationship is key for integral development, fostering empathy and solidarity.	Interdisciplinary collaboration and continuous training improve inclusion and support for students with special needs.
De Souza et al. [23]	2021	Duality of living with systemic lupus erythematosus (LES)	To explore the perceptions of people with SLE regarding their experience with the disease	Living with SLE involves facing emotional and physical ups and downs, highlighting the importance of professional, social, and family support.	Empathetic and comprehensive care significantly improves the quality of life of patients with chronic diseases like SLE.
Pelentsov et al. [11]	2015	The supportive care needs of parents caring for a child with a rare disease.	To identify the supportive care needs of parents caring for children with rare diseases	Main needs: social (72%), information (65%), and emotional (62%)	Importance of specific support strategies for families of children with rare diseases, including psychosocial and educational care
Mantovani et al. [14]	2018	Diagnosis and management of pseudo-hypoparathyroidism and related disorders.	To examine the diagnosis and treatment of pseudohypoparathyroidism (PHP) and related disorders	Multidisciplinary collaboration allows problems to be addressed throughout the life of patients with rare genetic diseases.	Emphasizes the need to implement patient registries and improve knowledge of the natural history of these diseases for better care.
Martínez et al. [24]	2020	Needs of caregivers and family members of individuals with orphan diseases based on a theoretical model.	To establish the needs of caregivers	Physical, psychological, social, educational, and economic needs were identified.	Nursing theory helps develop multidisciplinary interventions to care for caregivers, improving the quality of life for families.
Claahsen et al. [15]	2020	Current Knowledge on Congenital Adrenal Hyperplasia (CAH).	To provide updated information on advances in CAH and its management	Disease registries have increased data availability, highlighting psychosocial needs and advances in comprehensive management.	It is important for nursing professionals to update their knowledge to improve care for patients with rare diseases.
Medina-Ortega et al. [27]	2023	Clinical Case on Interstitial Pneumatosis.	To describe a rare clinical case to highlight approaches in nursing treatment	Adequate treatment allowed patient recovery, demonstrating the importance of individualized care in rare diseases.	Highlights the importance of specialized nursing knowledge for managing unusual clinical cases
Herbert et al. [25]	2021	Experience in Developing a Course for Nurses in the Care of Children with Genetic Diseases.	To describe the experience of developing a qualification course for nurses in the care of children with genetic diseases	The course covered the following topics: introduction to genetics in nursing; uncommon diseases; inborn errors of metabolism; neonatal screening program; and microcephaly.	Specialized training is crucial for addressing genetic diseases, promoting knowledge and leadership in nursing for treating these uncommon conditions.
Pleutim et al. [26]	2024	Person-Centered Care Practices for Individuals with Huntington’s Disease.	To explore care practices for people with Huntington’s disease from a patient- and family-centered approach	Families highlighted the need to adapt environments and actively seek knowledge about the disease to improve care. Specialized knowledge enables nurses to offer personalized and compassionate care to patients with rare diseases.	Online training modules on rare diseases were used, and participation in webinars and self-study modules reportedly increased the understanding of these pathologies by 78%.
Figueiredo et al. [19]	2020	Knowledge and Nursing Care Practices for Children with Pompe Disease in Intensive Care.	To examine nursing knowledge and practices in the care of children with Pompe disease in intensive care	Comprehensive care seeks to humanize care for children, highlighting the importance of specific knowledge of rare diseases.	Deep knowledge of the nursing team improves the quality and integration of intensive care in rare diseases like Pompe disease.
Gómez-Díaz et al. [20]	2023	Assessing knowledge, perceptions, awareness and attitudes on rare diseases among health care providers and health students in Mexico.	To evaluate the level of knowledge, perceptions, and attitudes of healthcare professionals and students in Mexico	A reliable questionnaire was administered to 200 healthcare professionals and students in Mexico to assess their level of knowledge, perceptions, awareness, and attitudes regarding rare diseases.	The study showed satisfactory knowledge levels and significant differences in perceptions, awareness, and attitudes among the study groups. The percentage of correct answers on a rare disease knowledge evaluation increased from 38% before the intervention to 93% after training.
Güner et al. [21]	2019	Survey Study Evaluating and Comparing Health Literacy Knowledge and Communication Skills Used by Nurses and Physicians.	To evaluate healthcare professionals’ knowledge and attitudes about health literacy, their communication skills, their effects on their practices, and to compare findings between healthcare professional subgroups	Most participants expressed willingness to receive information/training on the topic and to know the level of healthcare and whether it would change their approach and patient outcomes.	Both postgraduate and continuing education programs should be modified to improve the knowledge of all healthcare professionals and their positive effects on medical care.
Ferreira et al. [31]	2023	The utilisation of primary health care system concepts positively impacts the assistance of patients with rare diseases despite limited knowledge and experience by health care professionals: A qualitative synopsis of the evidence including approximately 78,000 individuals.	To compile available evidence on the impact of primary health care on patients with rare diseases and summarize published information from multiple stakeholders on the perceived usefulness and barriers to effective use of the primary care system	Primary healthcare teams are essential for guiding patients and families on emergency events. Technology-related concepts were reported in 19 publications, emphasizing their effectiveness in early diagnosis.	We provide valuable information on the effectiveness of primary healthcare in fostering a creative, inclusive, and supportive environment for patients with rare diseases. Our results can be useful for various stakeholders in deciding what actions are pending to achieve a solid and positive experience for patients with rare diseases in primary healthcare.
Morris et al. [28]	2022	Coordinated care for people affected by rare diseases: the CONCORD mixed-methods study.	To explore how care for people with rare diseases is coordinated in the UK and in what ways, and how people affected by rare diseases would like care to be coordinated	Care for people affected by rare diseases was found not to be well coordinated. For example, only 12% of 760 adult patients affected by a rare disease reported having a formal care coordinator, 32% reported attending a specialist center, and 10% reported having a care plan.	There is evidence of a lack of coordination in care for people affected by rare diseases, which can have negative impacts on the physical and mental health of patients and their families, as well as their economic well-being.
Czech et al. [12]	2020	A Review of Rare Disease Policies and Orphan Drug Reimbursement Systems in 12 Eurasian Countries.	To create a comprehensive and detailed overview of rare disease policies and MOH reimbursement in a selection of 12 countries in the Western Eurasian region: Armenia, France, Germany, Kazakhstan, Latvia, the Netherlands, Poland, Romania, Russia, Turkey, Ukraine, and the United Kingdom	The number of rare disease registries is increasing as a result of national plans (EU) and greater international scientific cooperation. Screening programs are widely implemented, but the number of diseases screened differs significantly (2–35 diseases), whether between EU and non-EU countries, among EU member states, and sometimes even within the same country.	Inequality in patient access to new OMPs still exists due to variations in national policies, healthcare budgets, health insurance, and reimbursement systems. The observed differences are a challenge for rare disease patients, health authorities, and manufacturers alike.
Tsitsani et al. [13]	2023	Barriers to and Facilitators of Providing Care for Adolescents Suffering from Rare Diseases: A Mixed Systematic Review.	To explore the barriers and facilitators to effective care for adolescents with rare diseases as perceived by patients and their parents	Institutional and public policy barriers were highlighted as the most frequently cited, resulting in unmet care needs and frustrating family dynamics.	National and regional rare healthcare policies are rarely truly linked to pragmatic intervention programs with a measurable impact on patient well-being.
Jahanshahi et al. [22]	2022	Iranian future healthcare professionals’ knowledge and opinions about rare diseases: cross-sectional study.	To investigate the knowledge and opinions of future Iranian healthcare professionals regarding rare diseases	Nearly 85% of participants rated their knowledge of rare diseases as poor or insufficient. Meanwhile, almost 70% of participants took courses on rare diseases at university.	A total of 72.7% of future healthcare professionals did not feel prepared to care for a patient with a rare disease. This highlights a gap in Iranian medical students’ knowledge of rare diseases.
Kamm et al. [32]	2020	Appendiceal Mucinous Neoplasm: Nurse Education About a Rare and Complex Disease.	To describe the diagnostic and treatment process for this disease, which is not well-known by most healthcare providers	Minimal information exists in the nursing literature on appendiceal mucinous neoplasm and the complexity of surgical treatment.	Nurses play an important role in the care of these patients and their specific needs, both pre- and post-surgery.
Pinto et al. [33]	2023	Implementation of genomic medicine for rare disease in a tertiary healthcare system: Mayo Clinic Program for Rare and Undiagnosed Diseases (PRaUD).	To integrate genomics into subspecialty practice, including specific genetic testing, research, and education	A total of 1152 patients were evaluated, with an overall resolution or probable resolution rate of 17.5%, and up to 66.7% depending on the phenotype. A total of 42.7% of patients with probable resolution underwent changes in medical treatment and outcomes.	The implementation of PRaUD (Program for Rare and Undiagnosed Diseases) and GTAC (Genomic Testing and Counseling) has allowed subspecialty practices to advance specialization in rare diseases, where historically genetic counselors have not been present.
Ward et al. [34]	2022	Designing rare disease care pathways in the Republic of Ireland: a co-operative model.	To explore the best approach to developing national rare disease care pathways within the Irish health system in the context of a lack of a consensual methodology	The roles of specialist nurse, psychology, medical social work, and database administrator were considered essential for all care pathways. Twenty-nine rare diseases across eighteen ERNs were selected for the development of care pathways.	Common needs of patients with renal diseases and interventions by healthcare professionals across all care pathways were identified. Key stakeholders in renal diseases have endorsed this national care pathway initiative.
Guccione et al. [29]	2019	Defining the Supportive Care Needs and Psychological Morbidity of Patients with Functioning Versus Nonfunctioning Neuroendocrine Tumors: Protocol for a Phase 1 Trial of a Nurse-Led Online and Phone-Based Intervention.	To describe the protocol for a study aimed at better understanding the outcomes and experiences of patients diagnosed with neuroendocrine tumors and to develop and test a nurse-led online and phone-based intervention that will provide personalized supportive care tailored to subgroups of patients with neuroendocrine tumors (functioning vs. nonfunctioning)	The limited research on patients with neuroendocrine tumors suggests that quality of life and patient experiences are significantly impaired compared to the general population.	The availability of disease-specific information was improved, as was the design of a nurse-led online and phone-based supportive care interventions tailored to the unique needs of the neuroendocrine tumor patient population.

## Data Availability

The data for this study is available upon request to the corresponding author.

## Public Involvement Statement

No public involvement in any aspect of this research.

## Guidelines and Standards Statement

This manuscript was drafted based on the PRISMA MODEL for systematic reviews.
